# Differences in Granule Morphology yet Equally Impaired Exocytosis among Cytotoxic T Cells and NK Cells from Chediak–Higashi Syndrome Patients

**DOI:** 10.3389/fimmu.2017.00426

**Published:** 2017-04-18

**Authors:** Samuel C. C. Chiang, Stephanie M. Wood, Bianca Tesi, Himmet Haluk Akar, Waleed Al-Herz, Sandra Ammann, Fatma Burcu Belen, Umran Caliskan, Zühre Kaya, Kai Lehmberg, Turkan Patiroglu, Huseyin Tokgoz, Ayşegül Ünüvar, Wendy J. Introne, Jan-Inge Henter, Magnus Nordenskjöld, Hans-Gustaf Ljunggren, Marie Meeths, Stephan Ehl, Konrad Krzewski, Yenan T. Bryceson

**Affiliations:** ^1^Center for Hematology and Regenerative Medicine, Department of Medicine, Karolinska Institutet, Karolinska University Hospital Huddinge, Stockholm, Sweden; ^2^Center for Infectious Medicine, Department of Medicine, Karolinska Institutet, Karolinska University Hospital Huddinge, Stockholm, Sweden; ^3^Childhood Cancer Research Unit, Department of Women’s and Children’s Health, Karolinska Institute, Karolinska University Hospital Solna, Stockholm, Sweden; ^4^Clinical Genetics Unit, Department of Molecular Medicine and Surgery, Center for Molecular Medicine, Karolinska Institutet, Karolinska University Hospital, Stockholm, Sweden; ^5^Faculty of Medicine, Department of Pediatric Immunology, Erciyes University, Kayseri, Turkey; ^6^Department of Pediatrics, Faculty of Medicine, Kuwait University, Kuwait City, Kuwait; ^7^Center for Chronic Immunodeficiency, University Medical Center Freiburg, University of Freiburg, Freiburg, Germany; ^8^Izmir Katip Celebi University Medical Faculty, Department of Pediatric Hematology and Oncology, Izmir Tepecik Training and Research Hospital, Izmir, Turkey; ^9^Meram Faculty of Medicine, Department of Pediatric Hematology, Necmettin Erbakan University, Konya, Turkey; ^10^Pediatric Hematology Unit of the Department of Pediatrics, Medical School of Gazi University, Ankara, Turkey; ^11^Department of Pediatric Hematology and Oncology, Division of Pediatric Stem Cell Transplantation and Immunology, University Medical Center Hamburg Eppendorf, Hamburg, Germany; ^12^Division of Pediatric Hematology and Oncology, Istanbul School of Medicine, Istanbul University, Istanbul, Turkey; ^13^Office of the Clinical Director, National Human Genome Research Institute, National Institutes of Health, Bethesda, MD, USA; ^14^Receptor Cell Biology Section, Laboratory of Immunogenetics, National Institute of Allergy and Infectious Diseases, National Institutes of Health, Rockville, MD, USA; ^15^Broegelmann Research Laboratory, Department of Clinical Sciences, University of Bergen, Bergen, Norway

**Keywords:** Chediak–Higashi syndrome, NK cells, cytotoxic T cells, exocytosis, lytic granule polarization, lysosomes, endosomes

## Abstract

Chediak–Higashi syndrome (CHS) is caused by autosomal recessive mutations in *LYST*, resulting in enlarged lysosomal compartments in multiple cell types. CHS patients display oculocutaneous albinism and may develop life-threatening hemophagocytic lymphohistiocytosis (HLH). While NK cell-mediated cytotoxicity has been reported to be uniformly defective, variable defects in T cell-mediated cytotoxicity has been observed. The latter has been linked to the degree of HLH susceptibility. Since the discrepancies in NK cell- and T cell-mediated cellular cytotoxicity might result from differences in regulation of cytotoxic granule release, we here evaluated perforin-containing secretory lysosome size and number in freshly isolated lymphocytes from CHS patients and furthermore compared their exocytic capacities. Whereas NK cells from CHS patients generally contained a single, gigantic perforin-containing granule, cytotoxic T cells predominantly contained several smaller granules. Nonetheless, in a cohort of 21 CHS patients, cytotoxic T cell and NK cell granule exocytosis were similarly impaired upon activating receptor stimulation. Mechanistically, polarization of cytotoxic granules was defective in cytotoxic lymphocytes from CHS patients, with EEA1, a marker of early endosomes, mislocalizing to lysosomal structures. The results leads to the conclusion that lysosome enlargement corresponds to loss of distinct organelle identity in the endocytic pathway, which on a subcellular level more adversely affects NK cells than T cells. Hence, vesicular size or numbers do not *per se* dictate the impairment of lysosomal exocytosis in the two cell types studied.

## Introduction

Chediak–Higashi syndrome (CHS, MIM 214500) is an autosomal recessive disease associated with mutations in *LYST* (1–3). *LYST* encompasses 55 exons and encodes an evolutionary conserved, ubiquitously expressed cytosolic protein of 3801 amino acids. CHS is characterized by oculocutaneous albinism and not seldom development of hemophagocytic lymphohistiocytosis (HLH), the latter a life-threatening hyperinflammatory syndrome (4, 5). However, age at onset of HLH is highly variable. Up to 20% of patients do not develop HLH. CHS patients may otherwise present with lymphoma (6, 7). Neurological deficits often appear in adolescence and may progress even after hematopoietic stem cell transplantation (8). An ability to predict clinical outcome based on genotype or diagnostic criteria, in particular with respect to HLH development, would be of value for the clinical management of CHS patients.

Orthologs of human LYST have been implicated in regulation of protein sorting and endosomal vesicle fusion (9–11). In humans, the defining characteristic of CHS is giant lysosomes and lysosome-related organelles (12). While this does not seem to impair the physiology of most cell types (12), functional defects are apparent in cells that perform lysosomal secretion, including melanocytes, lymphocytes, platelets, MHC class II-expressing antigen presenting cells, and glial cells, resulting in oculocutaneous albinism and prolonged bleeding (13). In lymphocytes, these defects lead to impaired cytotoxicity, a trait that has been closely linked to the development of HLH (7, 14–16). Development of lymphoma and an overall increased risk of cancer have also been reported in individuals with impaired lymphocyte cytotoxicity, including CHS patients (17–19). Mouse models have provided insights to the pathogenesis of CHS. In *souris* mice, which have a splice-site frameshift mutation in intron 27 of *Lyst*, development of HLH is seen upon LCMV infection (20). In contrast, *beige* mice, which have a 3-nucleotide in-frame deletion at the C-terminal encoding region of *Lyst*, do not develop HLH upon similar infection (1–3). A study encompassing analyses of mouse models as well as human CHS patients indicated that the risk of HLH development in CHS is determined by subtle differences in cytotoxic T lymphocyte (CTL) function, while NK cell cytotoxic function was generally more severely and uniformly compromised (20). While that study did not find clear genotype–phenotype correlations, other clinical studies have suggested genotype–phenotype correlations that may explain CHS severity (5).

To elucidate possible differences in T cell- and NK cell-mediated regulation of cytotoxic granule release in CHS, we here performed a detailed comparison of the characteristics and exocytic capacity of perforin-containing secretory lysosomes in cytotoxic lymphocyte subsets freshly isolated from CHS patients.

## Patients, Materials, and Methods

### Patients and Healthy Control Donors

Genetic analysis was subsequently performed as described below. As controls, peripheral blood was obtained from the Karolinska University Hospital blood bank.

### DNA Extraction, Amplification, and Sequence Analysis

Genomic DNA was isolated from the peripheral blood according to standard procedures. HLH-associated genes, including *LYST*, were sequenced as described previously (21). Mutations identified by high-throughput sequencing were verified by Sanger sequencing. Primers, PCR conditions, and sequencing reaction conditions are available on request.

### Cells

Peripheral blood was collected in heparin tubes and processed within 48 h of collection. Peripheral blood mononuclear cells (PBMC) were obtained by density gradient centrifugation (Lymphoprep; Axis-Shield) and resuspended in complete medium (RPMI 1640 medium with 10% FBS, l-glutamine, penicillin, and streptomycin; all Hyclone). For some experiments, NK cells were isolated from PBMC by negative selection (Miltenyi Biotec), maintained in complete medium, and used within 2 days of isolation. These cells were determined by flow cytometry to be more than 95% pure CD3^−^CD56^+^ NK cells. The human erythroleukemia cell line K562 and mouse mastocytoma cell line P815 (American Type Culture Collection) were maintained in complete medium.

### Antibodies

For cell staining and flow cytometry, fluorochrome-conjugated antibodies to CD3 (clone S4.1; Invitrogen), CD4 (S3.5; Invitrogen), CD8 (RPA-T8; BioLegend), CD16 (3G8; BD Biosciences), CD45 (30-F11; Invitrogen), CD56 (NCAM16.2; BD Biosciences), CD57 (HCD57; BioLegend), CD107a (H4A3; BD Biosciences), perforin (δG9; BD Biosciences), granzyme A (CB9; BD Biosciences), and granzyme B (GB11; BD Biosciences), were used. Fluorochrome-conjugated IgG1 and IgG2b (MOPC-21 and 27-35; BD Biosciences) isotype control antibodies were also used in addition to a fixable live/dead cell marker (Invitrogen). Mouse anti-CD3 (S4.1) and purified anti-CD16 (3G8) mAbs were used for redirected ADCC. For confocal microscopy, mouse monoclonal antibodies to α-tubulin (236-10501; Invitrogen), mannose-6-phosphate receptor (MEM-238; Invitrogen), and perforin (δG9; BioLegend) mAbs were used. Rabbit polyclonal antibodies used were Rab27a, Stx11, and Munc13-4 (all Protein Technologies Group), EEA1 (Cell Signaling Technologies), WASP-interacting protein (WIP, Santa-Cruz), and Cathepsin D (Upstate). Secondary antibodies were donkey anti-mouse and donkey anti-rabbit (both Invitrogen). DNA was labeled with DAPI and actin with phalloidin (both Invitrogen).

### Whole Blood Counts

Fifty microliters of fresh whole blood were stained in Trucount tubes (BD Biosciences) according to manufacturers instructions with fluorescent conjugated antibodies as listed above to distinguish various leukocyte and lymphocyte populations.

### Flow Cytometry and Functional Assays

Cells were either evaluated without pre-stimulation or after 36–48 h of stimulation with 500 IU/ml interleukin (IL)-2 (Proleukin, Novartis). For quantification of cytotoxic granule exocytosis, PBMC were mixed with target cells, with 2.5 μg/ml activating mAb where indicated as published (22). Cells were incubated for 2 h at 37°C. For intracellular staining, PBMC were surface stained with fluorochrome-conjugated antibodies as indicated. Cells were then fixed (Cytofix; BD Biosciences), followed by intracellular staining (Cytoperm; BD Biosciences). Isotype control antibodies were used as negative control. For assessment of functional responses, freshly isolated or IL-2-stimulated PBMC were incubated alone, with K562 cells, P815 cells, or P815 cells with either anti-CD16 or anti-CD3 antibody. After stimulation, the lymphocytes were surface stained with antibodies as indicated. All flow cytometry data were acquired on an LSRFortessa (BD Biosciences) and the resulting data were analyzed with FlowJo v9.9 software (TreeStar) and Prism Version 5.0 software (GraphPad). For analyses of cytotoxic granule constituent expression, at least 100 gated cytotoxic lymphocytes were analyzed.

Standard 4-h ^51^Cr-release assays were performed for NK cells as previously described (23). Briefly, 4 × 10^4^ ^51^Cr-labeled K562 target cells were incubated with peripheral blood effector cells in 200 μl of complete medium in 96-well V-bottom plates at various concentrations. Experiments were performed in triplicate, and effector to target cell ratios ranged from 100 to 0.3. The supernatants were measured for ^51^Cr release on a gamma-counter (Cobra Auto-Gamma, Packard). Lytic units at 25% target cell lysis were calculated as previously described (24). Cytotoxic T cell cytotoxicity requiring purified CD3^+^CD8^+^CD57^+^ cells was not performed due to the limited volumes of blood obtained from patients (22).

### Immunofluorescence and Confocal Microscopy

Freshly isolated NK cells or CTL, resting or stimulated with 100 ng/ml phorbol 12-myristate 13-acetate (PMA) (Calbiochem) and 0.5 mM ionomycin (Sigma-Aldrich), or co-incubated with K562 cells were spun down in glass-bottom plates (Matrical, Brooks). After 15 min, cells were fixed with 4% paraformaldehyde in PBS, permeabilized with 0.5% saponin in PBS, and blocked in PBS with 5% FBS, 0.1% BSA-c (Aurion), and 2% normal donkey serum (Jackson ImmunoResearch). Images were acquired on a confocal microscope (Nikon A1R) with a 63× oil objective, using NIS Elements Software, and analyzed using ImageJ (Research Service Branch, National Institutes of Health, Bethesda, MD) or PAD (Digital Cell Imaging Laboratories, Belgium). To quantify the size and number of lytic granules, mean fluorescence intensity, as well as distances between granules, immune synapses (IS) and the microtubule-organizing center (MTOC), images of fixed cells with were analyzed using the PAD software. Object detection based on perforin or CD107a labeling was performed automatically and checked manually. MTOC XY co-ordinates were selected manually based on α-tubulin labeling, and the center of the IS was entered based on phalloidin labeling and phase contrast images. Mean and standard deviation (SD) per patient were then graphed using Prism.

### Statistics

Statistical analysis was performed using Prism software (version 5, GraphPad Software, Inc.), as specified.

## Results

### Characteristics of CHS Patients

In characterizing this cohort of CHS patients, we identified biallelic mutations in *LYST* in a total of 21 patients from 14 unrelated families (Table 1). While the majority of identified *LYST* mutations have been previously described (20, 21, 25–30), *LYST* c.3938delA (p.Q1313RfsX4) in family 9, and c.265insA (p.R886TfsX5) and c.5601delA (p.K1867NfsX11) in family 12 are novel. With the exception of *LYST* p.A1454N (patient 19) and p.G408R (families 17 and 18), which were present in compound heterozygous form, all mutations were nonsense or frameshift mutations predicted to result in truncated LYST protein. Clinically, 20 out of 21 patients had oculocutaneous albinism, 11 out of 21 patients developed HLH, and 12 out of 21 patients suffered neurological sequelae, including nystagmus, convulsions, and abnormal MRI, The six adult patients who had not developed HLH displayed albinism and neurological symptoms. Only three patients were transplanted, all successfully.

**Table 1:** **Clinical and laboratory findings in patients with *LYST* mutations**.

Patient code	1	2	3	4	5	6	7	8	9	10	11
Mutation	c.1540C>T; p.R514X (hmz)	c.1902dup; p.A635SfsX4 (hmz)	c.1902dup; p.A635SfsX4 (hmz)	c.2311C>T; p.Q771X (hmz)	c.2749_50delAG; p.R917GfsX5 (hmz)	c.2749_50delAG; p.R917GfsX5 (hmz)	c.2749_2750del: p.R917GfsX5 (hmz)	c.2749_2750del: p.R917GfsX5 (hmz)	c.3938delA; p.Q1313RfsX4 (hmz)	c.4508C>G; p.S1483X (hmz)	c.5506C>T. p.R1836X (hmz)
Mutation citation	Zarzour et al. (30)	Karim et al. (27)	Karim et al. (27)	Tesi et al. (18)	Tesi et al. (18)	Tesi et al. (18)	Tesi et al. (18)	Tesi et al. (18)	This report	Jessen et al. (20)	Kaya et al. (29)
Protein domain	Unspecified	N-term Arm/HEAT	N-term Arm/HEAT	N-term Arm/HEAT	N-term Arm/HEAT	N-term Arm/HEAT	N-term Arm/HEAT	N-term Arm/HEAT	77aa Upstream of ConA	ConA-like domain	145aa ds of ConA
Country of origin	Turkey	Kuwait	Kuwait	Kuwait	Turkey	Turkey	Turkey	Turkey	Turkey	Egypt	Turkey
Familial disease	−	−	−	−	+ (sibling)	+ (sibling)	+ (sister)	+	−	ND	+ (sibling)
Parental consanguinity	+	+	+	+	+	+	+	+	+	+	+
Sex	Female	Female	Male	Female	Male	Female	Female	Female	Female	Male	Female
Age at hemophagocytic lymphohistiocytosis (HLH) diagnosis	4 years	None, 5 years	18 months	2 years	2 years	None, 11 years	3 years	None, 15 moths	2 years	4.5 years	4 years
Fever	+	−	+	+	+	−	ND	ND	+	+	+
Splenomegaly	+	−	+	+	+	−	+	+	+	+	+
Hepatomegaly	+	−	+	+	+	−	+	+	+	+	+
Hemoglobin (g/l)	60	ND	78	65	79	ND	ND	ND	110	<90	85
Neutrophils (10^9^/l)	0.8	ND	0.7	0.35	3.8	ND	1.2	ND	0.77	<1	0.14
Platelets (10^9^/l)	8	ND	90	35	48	ND	ND	ND	146	ND	24
Triglycerides (mmol/l)	2.3	ND	5.3	8.1	5.5	ND	ND	ND	ND	ND	3.2
Fibrinogen (g/l)	0.8	ND	1.5	2.5	ND	ND	ND	ND	ND	ND	0.5
Hemophagocytosis	+	ND	−	−	ND	ND	ND	ND	ND	+	+
Ferritin (μg/l)	1,350	ND	3,600	1,151	1,482	ND	ND	ND	949	ND	2,000
sCD25 (U/ml)	ND	ND	ND	ND	1,844	ND	ND	ND	ND	−	ND
Neurological sequelae^§^	−	−	−	+	ND	ND	+	−	ND	ND	+
Oculocutaneous albinism	+	+	+	+	+	ND	+	+	+	+	Atypical
Pathological CSF	−	ND	ND	−	ND	ND	ND	ND	ND	ND	+
Infection	EBV	−	CMV, EBV	−	ND	ND	Recurrent respiratory infect	Recurrent respiratory infect.	ND	ND	*Salmonella*
Treatment active disease	HLH-2004	−	Steroids/IVIG	HLH-2004	HLH-2004?	ND	ND	NA	−	ND	HLH-2004
Remission at 2 months	+	NA	+	+	ND	ND	NA	NA	NA	+	+
Age at HSCT	Scheduled	NA	NA	NA	NA	ND	NA	NA	NA	ND	NA
Outcome	Alive	Alive	Alive	Deceased	ND	ND	Deceased 3 years AML	Alive	Lost to follow up	ND	Deceased

**Patient code**	**12**	**13**	**14**	**15**	**16**	**17**		**18**	**19**	**20**	**21**

Mutation	c.2656insA; p.R886TfsX5 Ex8 + c.5601delA; p.K1867NfsX11	IVS19 c.5784 + 5G>T splice donor site (hmz)	c.9107-20_9109 delTATCCAATTACTTTCTGCAGGTA (hmz)	c.9827_9832ATACAA; p.N3276_T3277del (hmz)	c.9827_9832ATACAA; p.N3276_T3277del (hmz)	c.3310C>T; p.R1104X + c. 10222G>A; p.G3408R		c.3310C>T; p.R1104X + c. 10222G>A; p.G3408R	c.4361C>A; p.A1454D + c.5061T>A; p.Y1687X	c.4361C>A; p.A1454D + c.5061T>A; p.Y1687X	c.2570C>G; p.S857C + c.9930delT; p.F3310LfsX36
Mutation citation	This report	Jessen et al. (20)	Certain et al. (26)	Weisfeld-Adams et al. (28)	Weisfeld-Adams et al. (28)	1104X: Nagle et al. (2) Gly3408Arg: this report		1104X: Nagle et al. (2) Gly3408Arg: this report	Karim et al. (27)	Karim et al. (27)	Jessen et al. (20)
Protein domain	N-term Arm/HEAT	ds of ConA	WD-40 repeat domain	BEACH	BEACH	Arm/HEAT, BEACH		Arm/HEAT, BEACH	ConA	ConA	Arm/HEAT point, BEACH
Ethnic origin	Germany	Kuwait	Turkey	Pakistan	Pakistan	USA		USA	USA	USA	Turkey
Familial disease	−	−	+	+	+	+		+	+	+	ND
Parental consanguinity	−	+	+	+ (distant)	+ (distant)	−		−	−	−	ND
Sex	Male	Female	Male	Male	Female	Male		Male	Male	Male	Male
Age at diagnosis-HLH	11 years	7 years	11 months	None, 34 years	None, 44 years	None, 28 years		None, 23 years	None, 31 years	None, 28 years	2 years
Fever	+	+	+	−	−	−		−	−	−	+
Splenomegaly	+	+	+	−	−	+		−	+	+	?
Hepatomegaly	+	+	+	−	−	+		−	Upper Nl	+	+
Hb (g/l)	77	83	58	139	119	144		150	159	144	79
Neutrophils (10^9^/l)	ND	3.5	0.4	1.88	2.65	3.13		1.85	0.69	1.12	0.3
Platelets (10^9^/l)	40	75	38	249	357	275		251	118	182	47
Triglycerides (mmol/l)	3.8	4.01	4.4	1.37	1.72	1.85		0.67	1.04	2.31	7.8
Fibrinogen (g/l)	0.8	2.3	0.12	5.22	4.14	4.32		2.91	2.62	2.70	1.0
Hemophagocytosis	−	−	+	−	−	−		−	−	−	−
Ferritin (μg/l)	7,931	1,500	2,000	85	87	155		159	95	67	2,405
sCD25 (U/ml)	32,366	ND	1,500	268	498	527		374	415	521	32,565
Neurological sequelae^§^	+	−	+	+	+	+		+	+	+	+
Oculocutaneous albinism	+	+	+	+	Minimal	+		+	+	+	+
Pathological CSF	−	−	Parenchymal involvement without CSF	ND	ND	ND		ND	ND	ND	+
Infection	−	EBV	Pneumonia	−	−	−		−	Skin abscess	Skin abscess	EBV
Treatment active disease	+	HLH-2004	Etoposide, IVIg, CycloA, Dexamethasone	−	−	−		−	−	−	+
Remission at 2 moths	+	+	+	NA	NA	NA		NA	NA	NA	+
Age at HSCT	11 years	8 years	NA	NA	NA	NA		NA	NA	NA	2 years
Outcome	Alive	Alive	Deceased	Alive	Alive	Alive		Alive	Alive	Alive	Alive

*Values for HLH parameters are from time of diagnosis. §: Reported at some point during the course of the disease, N/A: not applicable, N/D: no data*.

### Normal Cytotoxic Lymphocyte Numbers in Peripheral Blood of CHS Patients

**Figure 1:**
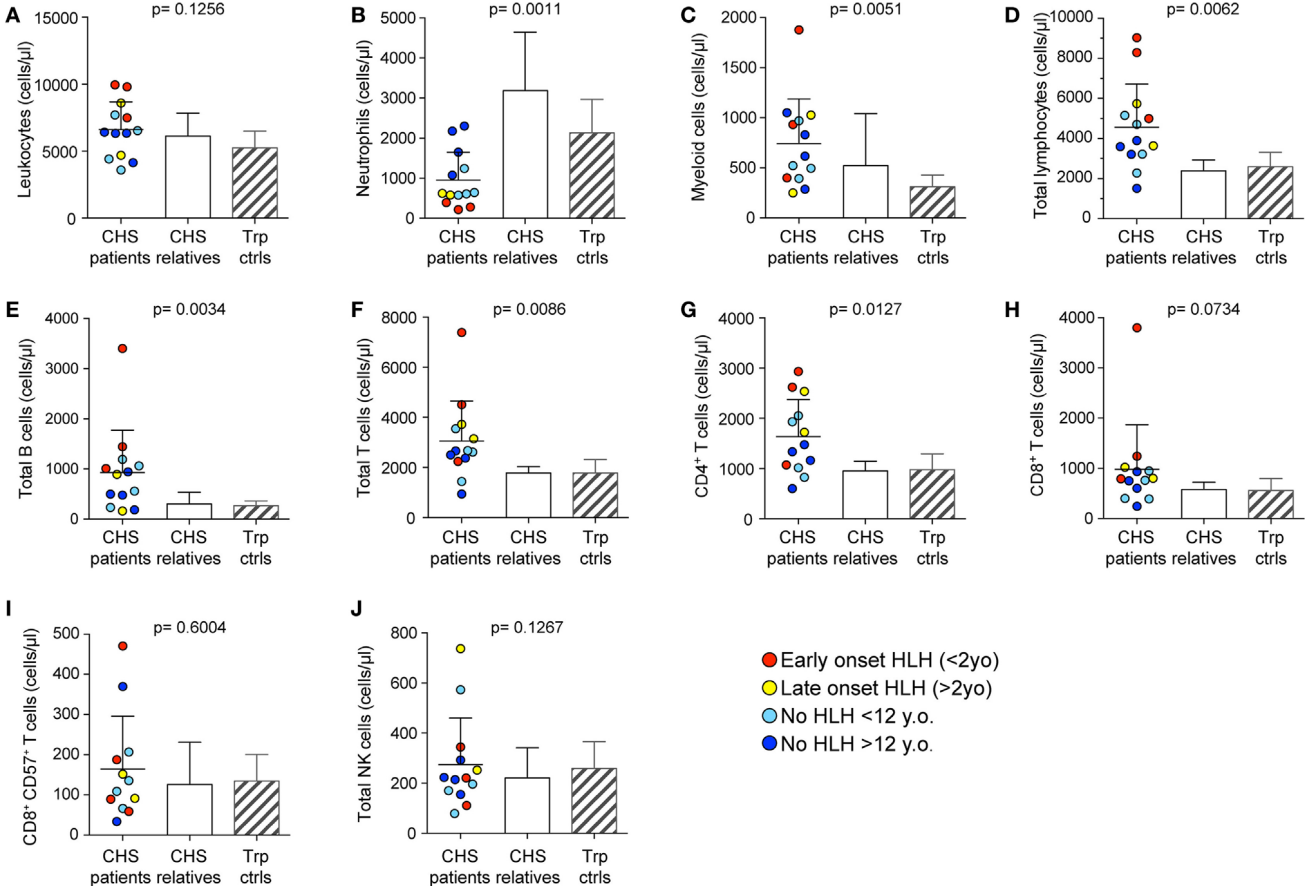
**Peripheral blood cell numbers in Chediak–Higashi syndrome (CHS) patients**. **(A)** Total leukocyte, **(B)** neutrophil, **(C)** monocyte, **(D)** lymphocyte, **(E)** CD19^+^ B cell, **(F)** CD3^+^ T cell, **(G)** CD3^+^CD4^+^ T cell, **(H)** CD3^+^CD8^+^ T cell, **(I)** cytotoxic CD3^+^CD8^+^CD57^+^ T cell, and **(J)** bulk CD3^−^CD56^+^ NK cell numbers were enumerated in peripheral blood from 13 CHS patients. The patients are color-coded according to whether they presented with early-onset hemophagocytic lymphohistiocytosis (HLH) (<2 years), late-onset HLH (>2 years), or no HLH diagnosis, as indicated. Patient cell numbers were compared to those of healthy relatives and transport controls, as indicated. Columns depict mean values, bars indicate SD. Non-parametric one-way ANOVA Kruskal–Wallis tests are reported as exact *p* values.

Peripheral blood from the 21 CHS patients with defined mutations in *LYST* were analyzed with respect to numbers and also stratified according to age at onset of HLH (Table 1). To grossly assess whether the CHS patient cytotoxic lymphocytes developed and differentiated normally, leukocyte subsets were enumerated in whole blood from 13 patients. In this respect, we examined PBMC counts of patients and compared them with healthy related (family) and unrelated (transport control) samples. CHS patient leukocyte numbers were similar to numbers observed in healthy individuals (Figure 1A). More specifically, neutrophil numbers were significantly decreased in CHS patients (Figure 1B), whereas monocyte cell numbers tended to be higher in CHS patients (Figure 1C), and lymphocyte numbers were significantly elevated in CHS patients as compared to healthy controls (Figure 1D). Overall B cell and T cell numbers were elevated above both control groups (Figures 1E–G), while the CD8^+^ T cell subset was not specifically elevated (Figure 1H). With respect to cytotoxic lymphocytes, CTL (CD8^+^CD57^+^ T cells) and NK cell numbers were not significantly elevated in CHS patients (Figures 1I,J), in contrast with a previous report that indicated somewhat elevated HNK-1^+^ (CD57^+^) cell numbers in CHS patients (31). The severity of lymphocytosis, consisting of elevated B cell and CD4^+^ T cell numbers, and neutropenia was most severe in patients with early onset HLH, and least severe in patients with no HLH (data not shown).

### Expression of Granule Constituent Proteins in CHS Patient Cytotoxic Lymphocytes

**Figure 2:**
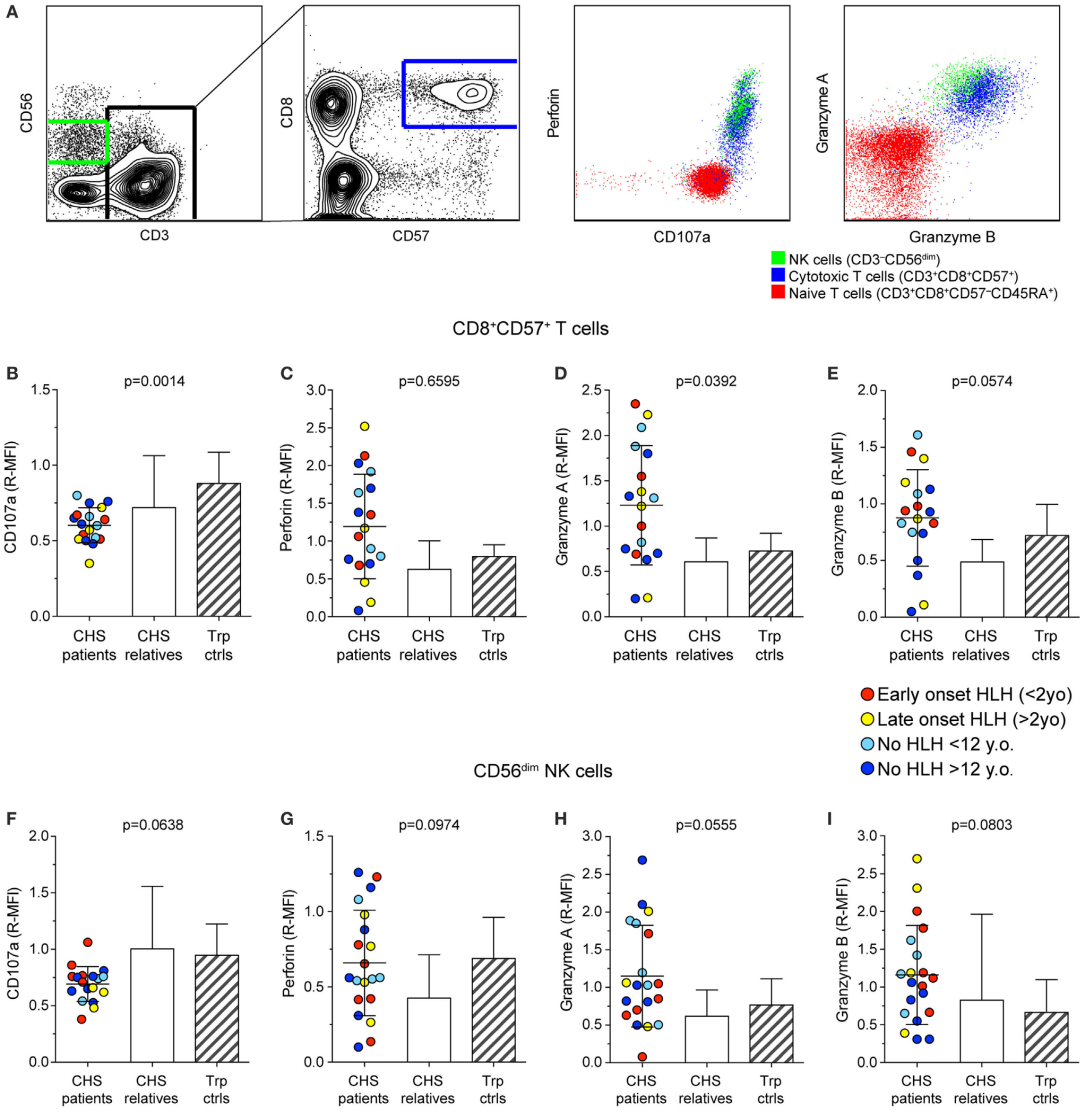
**Expression of granule constituent proteins in cytotoxic lymphocyte subsets of Chediak–Higashi syndrome (CHS) patients**. Gating strategy and representative dot plots of various lysosomal markers are shown in **(A)**. Peripheral blood mononuclear cells were labeled for intracellular granule constituent proteins as well as surface proteins to distinguish [**(B–E)**; *n* = 18 patients] CD3^+^CD8^+^CD57^+^ cytotoxic T cell and [**(F–I)**; *n* = 20 patients] CD3^−^CD56^dim^ NK cells, as indicated. Plots depict relative median fluorescent intensities of **(B,F)** CD107a, **(C,G)** perforin, **(D,H)** granzyme A, and **(E,I)** granzyme B in CHS patients, healthy relatives and transport controls, as indicated. The patients are color-coded according to whether they presented with early-onset HLH (<2 years), late-onset HLH (>2 years), or no HLH diagnosis, as indicated. Columns depict mean values, bars indicate SD. Non-parametric one-way ANOVA Kruskal–Wallis tests are reported as exact *p* values.

To examine whether CHS patient cytotoxic lymphocytes contained sufficient cytotoxic cargo proteins to induce target cell killing, we labeled freshly isolated PBMC with fluorochrome-conjugated antibodies to surface lineage and differentiation markers as well as to intracellular cytotoxic granule proteins and analyzed cells by flow cytometry (Figure 2A). The median fluorescence intensity of CD107a (lysosomal-associated membrane protein 1), perforin, granzyme A, and granzyme B was measured in CD3^+^CD8^+^CD57^+^ CTL (Figures 2B–E) and CD3^−^CD56^dim^ NK cell (Figures 2F–I) subsets in 18 and 20 patients, respectively. Values from patients, as well as familial and transport controls were normalized against local controls. Intracellular CD107a expression was slightly reduced, whereas perforin expression in CTL and NK cells varied considerably among patients. Granzyme A and B expression was elevated in patient CTL and NK cells. No correlation between expression of cytotoxic granule constituents and HLH diagnosis or age was observed (Figure 2). Thus, consistent with published analyses on 14-day cultured CHS CTL clones that indicated normal perforin and granzyme expression and processing (32), freshly isolated CHS patient cytotoxic lymphocytes expressed normal levels of cytotoxic granule constituents. If anything, levels of perforin and granzymes A and B were elevated in CHS patient lymphocytes, as observed in other familial HLH patients with mutations in genes required for lymphocyte cytotoxic granule exocytosis (23, 33). Of note, granzymes play pleiotropic roles in immune responses, not restricted to induction of target cell death (34).

### Cytotoxic Granule Morphology Differs between NK Cells and Cytotoxic T Cells in CHS Patients

**Figure 3:**
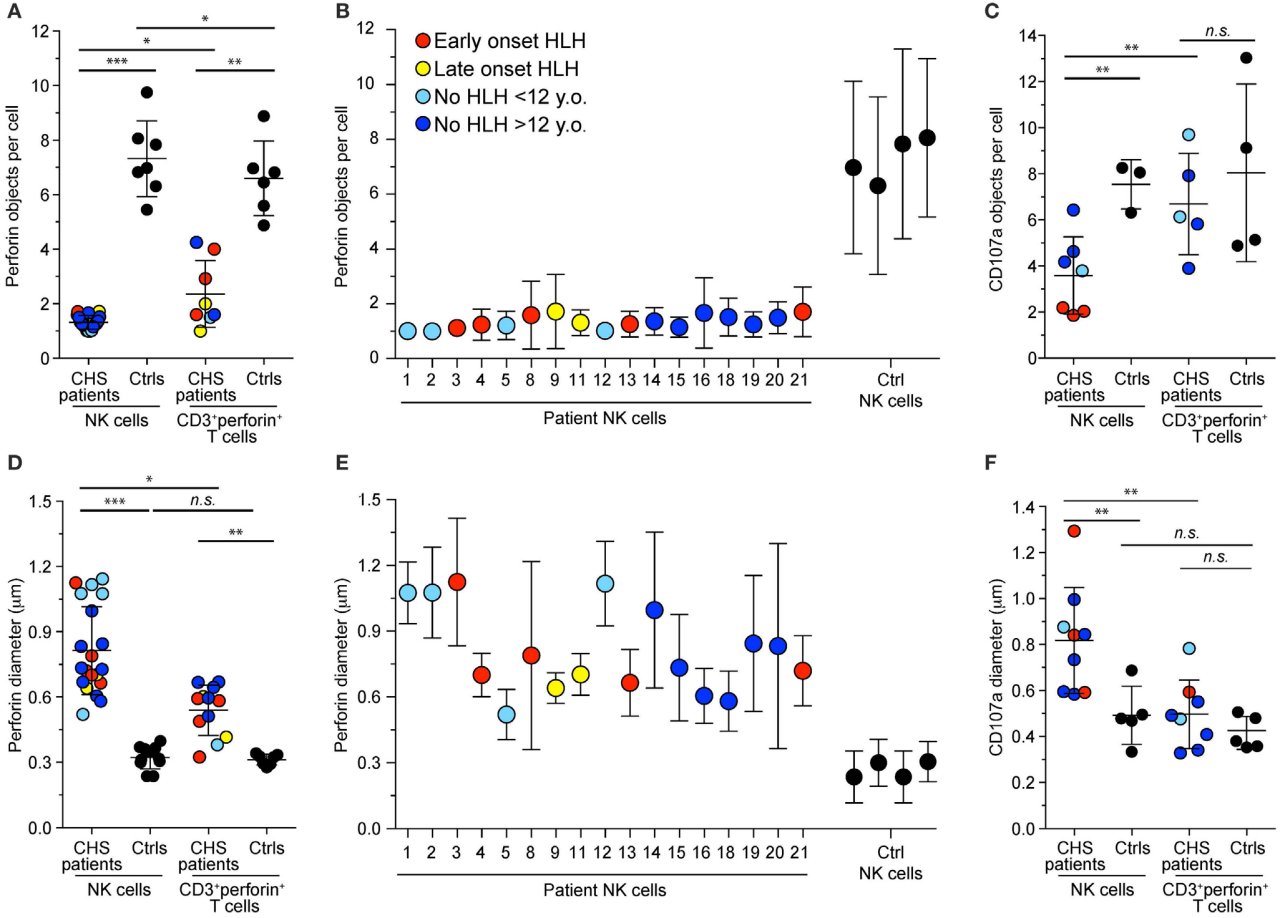
**Cytotoxic granule numbers and size in NK and T cells of Chediak–Higashi syndrome (CHS) patients**. Freshly isolated, blood-derived NK cells and CD3^+^perforin^+^ T cells were labeled intracellularly with antibodies to perforin and CD107a. Plots depict the **(A–C)** number and **(D–E)** diameter of **(A,B,D,E)** perforin or **(C,F)** CD107a granules per NK cell or cytotoxic T lymphocyte from individual CHS patients and healthy controls. Values represent the average of multiple cells in each individual with mean and SD indicated for **(A,C,D,F)** cumulated individuals or for **(B,E)** each patient and control individually. Symbols represent mean values and bars SD. With respect to perforin stainings, an average of 69 (range 4–510) CHS patient and 176 (range 35–536) control NK cells and 7 (range 2–14) CHS patient and 144 (range 35–309) control T cells and ere quantified. The patients are color-coded according to whether they presented with early-onset hemophagocytic lymphohistiocytosis (HLH) (<2 years), late-onset HLH (>2 years), or no HLH diagnosis, as indicated. Non-parametric Mann–Whitney U tests were performed.

Lysosomes from all cell types are enlarged in CHS patients and mouse models carrying *Lyst* mutations (35). We assessed lysosomal size and number in freshly isolated NK cells and CTL from our cohort of CHS patients by immunofluorescence staining of perforin and CD107a and analyses by confocal microscopy. The number of perforin (Figures 3A,B) and CD107a (Figure 3C) objects and their diameters (Figures 3D–F) among CTL (defined as CD3^+^perforin^+^ cells) or NK cell in patients and healthy controls (both familial and unrelated transport samples combined) were determined, providing estimates of perforin dense core numbers and diameters, as well as diameters of the outer, CD107a-coated cytotoxic granule limiting membrane (36). It should be noted that it has previously been reported that CD57^+^ NK cells show a particularly striking phenotype, with a single large cytotoxic granule (31), but lysosomal size and numbers have not been systematically compared between CTL and NK cells. In our cohort, the three patients (patients 1, 2, and 3) with the most 5′ *LYST* nonsense mutations had just one, enlarged perforin-containing vesicle in 81, 98, and 100% of NK cells, respectively. NK cells from 17 patients in total were examined and found to have 1–2 perforin-containing vesicles per cell (1.3 vesicles on average), whereas CTLs from seven patients had 1–4 perforin-containing vesicles per cell (3.6 vesicles on average). As such, relative to NK cells, T cells from CHS patients had significantly more cytotoxic granules (Figure 3A). In contrast, both NK cells and CTL from healthy controls averaged approximately seven perforin-containing vesicles per cell (Figure 3A). By comparison, CHS patients showed more numerous CD107a-delimited vesicles in NK cells compared to CTL (Figure 3C). Reflecting enlarged cytotoxic granules, in the three patients with the earliest *LYST* nonsense mutations, the mean NK cell perforin object diameter was 1.1 μm (0.81 ± 0.20, mean ± SD, for all 17 patients examined), with a CD107a object diameter of up to 1.3 μm (0.82 ± 0.23 μm), significantly larger than perforin (0.33 ± 0.04 μm) and CD107a (0.49 ± 0.13 μm) object diameters in normal NK cells (Figures 3D–F). CTL from the same patients contained more than one perforin object of a smaller size (0.50 ± 0.15 μm) relative to CD107a objects (0.54 ± 0.12 μm), albeit significantly larger than control CTL perforin (0.31 ± 0.03 μm) and CD107a (0.42 ± 0.72 μm) object diameters. The larger diameter of CD107a relative to perforin objects likely reflects staining of perforin in dense cores, whereas CD107a is confined to outer, delimiting membranes (36). In several patients, lysosomal morphology was also measured following 20 min of stimulation with target cells or PMA and ionomycin without any discernable difference observed in size or number of cytotoxic granules per cell (data not shown). Altogether, in spite of overall perforin expression being higher in NK cells relative to CTL (22), CHS patient NK cells most often had one giant perforin-containing cytotoxic granule as opposed to smaller and more numerous cytotoxic granules in CTL.

### Exocytosis is Equally Impaired in CHS Patient CTL and NK Cells

**Figure 4:**
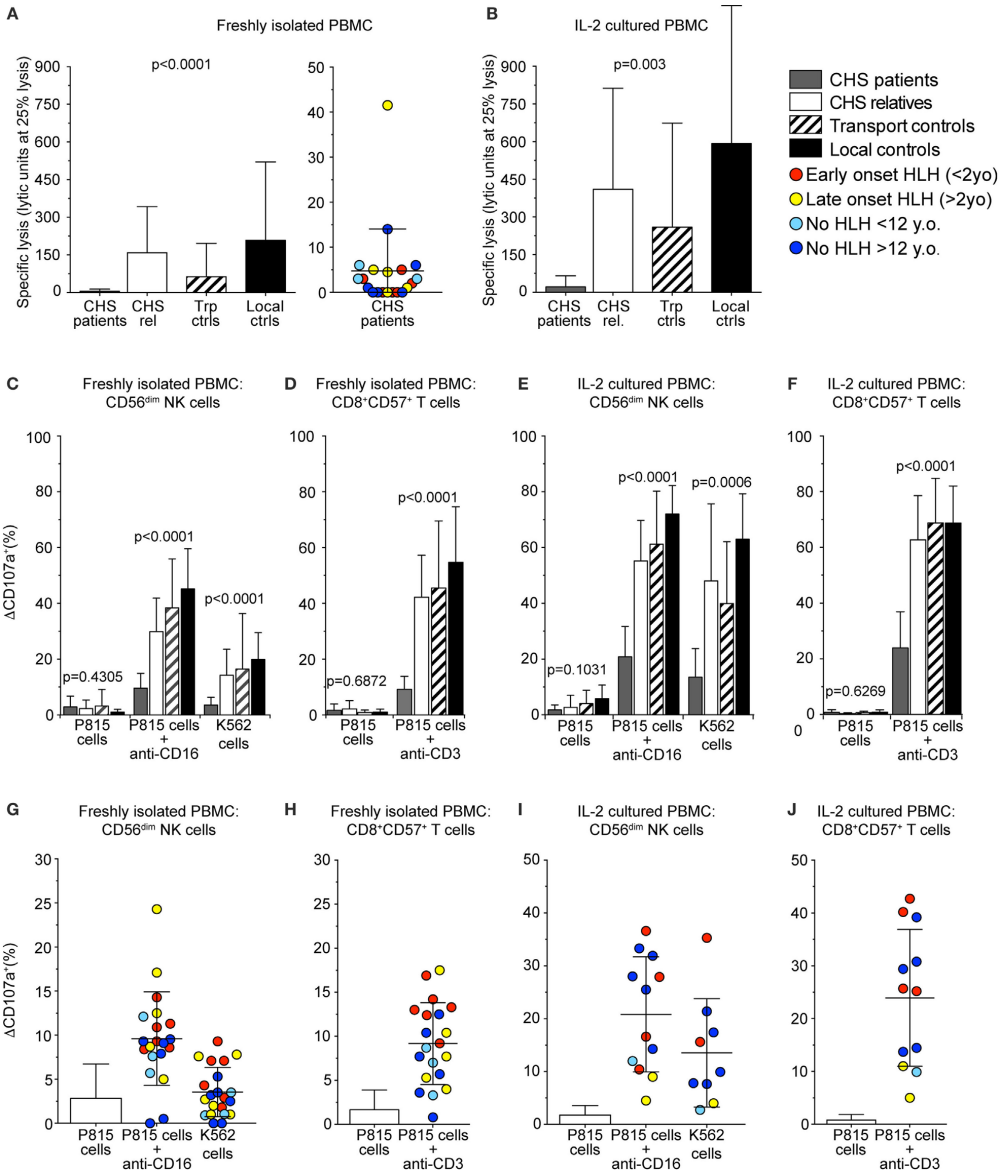
**Chediak–Higashi syndrome (CHS) patient NK and T cells have defective cytotoxicity and exocytosis**. Freshly isolated peripheral blood mononuclear cells (PBMCs) or PBMCs stimulated with interleukin (IL)-2 for 36–48 h from healthy family members, transport controls, and local Swedish controls were evaluated for cytotoxicity and exocytosis toward target cells. **(A,B)** Plots depict NK cell-mediated K562 target cell lysis by **(A)** freshly isolated or **(B)** IL-2 stimulated PBMC. **(C–J)** CD107a surface expression was assessed by flow cytometry after stimulation of PBMC with P815 cells alone, P815 cells supplemented with anti-CD16 or anti-CD3 antibody, or K562 cells, as indicated. Plots depict induced CD107a surface expression as quantified on gated **(C,E,G,I)** CD56^dim^ NK cells or **(D,F,H,J)** CD8^+^CD57^+^ CTL, **(C,D,G,H)** freshly isolated, or **(E,F,I,J)** stimulated with IL-2. **(A–F)** Columns represent mean values and bars SD. **(G–J)** Symbols represent mean values and bars SD. The patients are color-coded according to whether they presented with early-onset hemophagocytic lymphohistiocytosis (HLH) (<2 years), late-onset HLH (>2 years), or no HLH diagnosis, as indicated. Non-parametric one-way ANOVA Kruskal–Wallis tests are reported as exact *p* values.

For release of cytotoxic granule content and target cell killing, perforin-containing granules traverse the actin cytoskeleton (37, 38). Smaller cytotoxic granules in CTL might, therefore, enable more efficient traversation and exocytosis. Based on this notion and previous findings of more granule exocytosis by *in vitro* stimulated CTL as compared to freshly isolated NK cells (20), we thus assessed whether freshly isolated or stimulated CTL from CHS patients might display more cytotoxic granule exocytosis than NK cell counterparts. Lysis of K562 target cells by freshly isolated PBMC was defective compared with controls and below the limit set as pathologic for a diagnosis of HLH in all except patients 6 and 15 (Figure 4A) (39). Culturing healthy control PBMC in IL-2 for 36–48 h increased NK cytotoxicity against K562 cells. CHS patient NK cell cytotoxicity also improved with IL-2, as previously shown (40), but not to the level seen in familial controls (Figure 4B), or patients with *STX11* mutations (14, 23).

Cytotoxic lymphocyte exocytosis was assessed in PBMC co-incubated with either K562 or P815 cells added anti-CD16 antibody to trigger NK cells, or P815 cells added anti-CD3 to trigger CTL (Figures 4C–J). Exocytosis induced by K562 cells was defective (ΔCD107a^+^ < 5%) in CD3^−^CD56^+^ NK cells (ΔCD107a^+^ 3.5 ± 2.8%; mean ± SD) in 15 of 20 CHS patients (Figures 4C,G). Exocytosis induced by P815 cells added anti-CD16 was reduced in CD3^−^CD56^+^ NK cells from CHS patients (ΔCD107a^+^ 9.6 ± 5.3%), relative to healthy controls (ΔCD107a^+^ 45.2 ± 14.4%), though higher than against K562 cells (Figures 4C,G). Exocytosis induced by P815 cells added anti-CD3 was defective in CD3^+^CD8^+^CD57^+^ CTL (ΔCD107a^+^ 9.2 ± 4.6%), relative to healthy controls (ΔCD107a^+^ 54.7 ± 19.9%, Figures 4D,H). No correlation was observed between age of onset of HLH and the degree of impairment of fresh or short-term stimulated NK cell or CTL degranulation (not shown). Neither did the severity of the mutation correlate with exocytosis in freshly isolated nor IL-2 activated NK or T cells (Figures 4E,F,I,J), as was previously observed (20). Thus, contrary to our expectations, our assays did not uncover evidence supporting the notion of differential impairment of cytotoxic granule exocytosis in CTL versus NK cells from CHS patients.

### Freshly Isolated CHS Patient NK Cells form Immunological Synapses with Target Cells, but Fail to Polarize Granules to the Synapse

**Figure 5:**
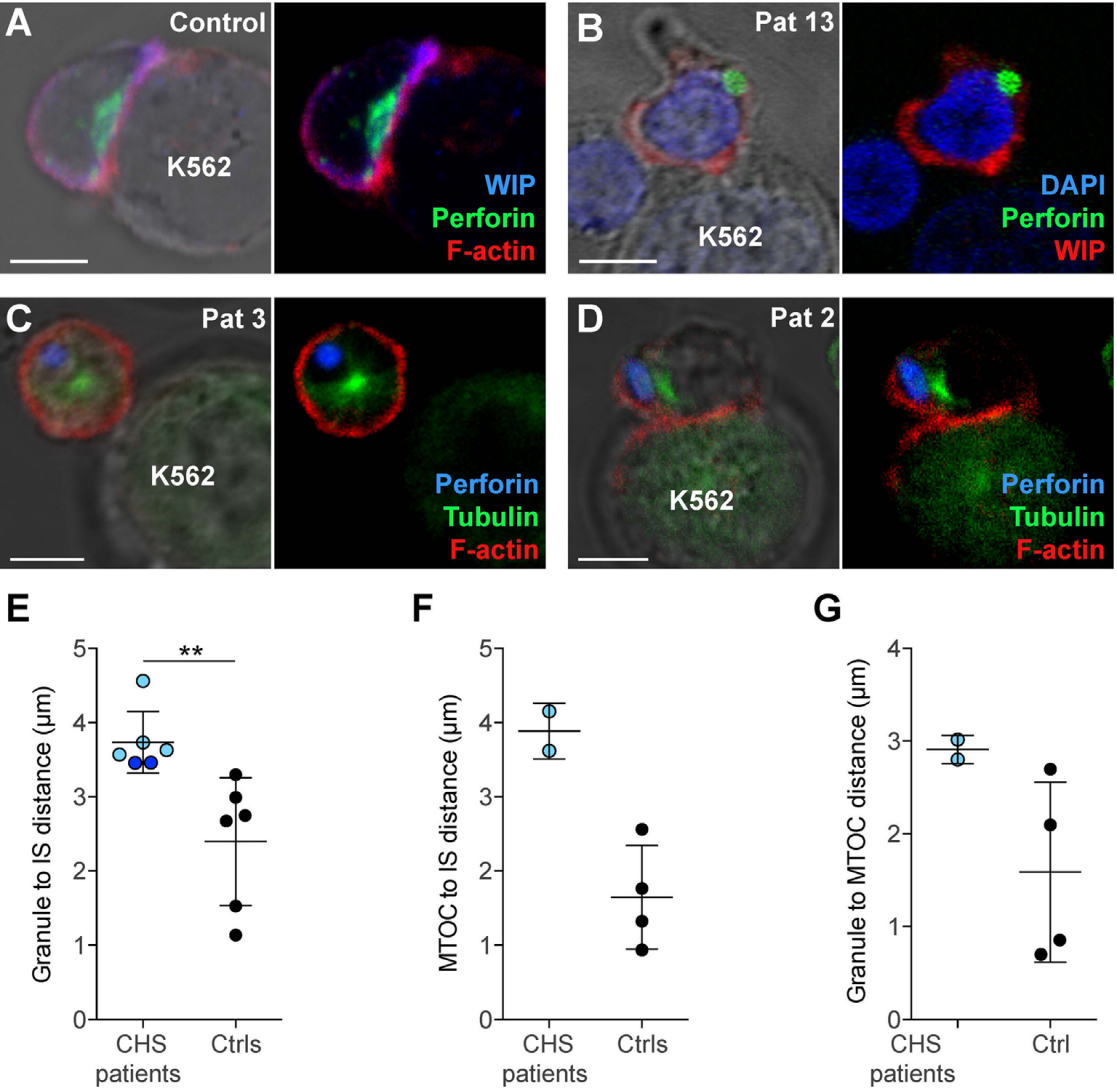
**Chediak–Higashi syndrome (CHS) patient NK cells form conjugates with target cells, but granules and microtubule-organizing center (MTOC) do not efficiently polarize to the immune synapse (IS)**. **(A)** A representative NK cell from a healthy donor conjugated to a K562 target cell, labeled with antibodies to WASP-interacting protein (WIP) and perforin as well as phalloidin to detect F-actin, as indicated. **(B)** A representative NK cell from a CHS patient conjugated to a K562 cell, labeled with antibodies to WIP and perforin, in addition to DAPI to discern the nucleus, as indicated. **(C–D)** NK cells from CHS patients conjugated to K562 cells, labeled with antibodies to perforin and tubulin as well as phalloidin to detect F-actin, as indicated. **(A–D)** Scale bars indicate 5 μm. Distances were measured between **(E)** MTOC and IS, **(F)** granules and IS (center of the contact between NK cell and target cell), or **(G)** granule and MTOC. **(E–G)** Each symbol represents average values from multiple cells from individual patients and controls, with overall mean and SD indicated. **(E)** 45 (range 9–150) CHS patient and 266 (range 54–488) control NK cells and **(F,G)** 17 (range 16–18) CHS patient and 269 (range 201–325) control NK cells were quantified.

In order to assess cytotoxic IS formation and granule polarization, freshly isolated NK cells from two CHS patients were mixed with K562 targets and allowed to form conjugates, which were then analyzed by microscopy. Healthy control NK cells conjugated to K562 target cells polarized perforin granules and accumulated actin and WIP, a key mediator of cytotoxic granule polarization and cellular cytotoxicity (41), at the mature IS (Figure 5A). NK cells from CHS patients were able to form conjugates with K562 targets to a similar extent as healthy controls and showed accumulated actin and WIP at the synapse, demonstrating that a mature IS was formed (Figures 5B–D). Strikingly, CHS NK cells failed to polarize their perforin granules to the mature IS (Figures 5E–G). The distance between the perforin granule and the IS in CHS NK cells was markedly increased compared with healthy donors (Figure 5E; 3.7 ± 0.42 μm, mean ± SD, versus 2.4 ± 0.86 μm, respectively). Similarly, the average distance of the MTOC to the synapse also was increased in LYST-deficient NK cells relative to controls (Figure 5F; 3.9 ± 0.38 μm in CHS patients versus 1.7 ± 0.69 μm in controls). Furthermore, the distance of the granules to the MTOC was also increased in LYST-deficient NK cells relative to controls (Figure 5G; 2.9 ± 0.15 versus 1.6 ± 0.97 μm, respectively). This indicates that in freshly isolated, LYST-deficient NK cells, cytotoxic granule coalescence and MTOC trafficking, early events in cytotoxic granule secretion, are severely impaired. Together, these results substantiate observations in IL-2 cultured NK cells and T cells from CHS patients, where normal IS formation yet impaired cytotoxic granule trafficking has been reported (25, 42).

### Enlarged Lysosomes in CHS NK Cells Colocalize with Early and Late Endosomal Markers

**Figure 6:**
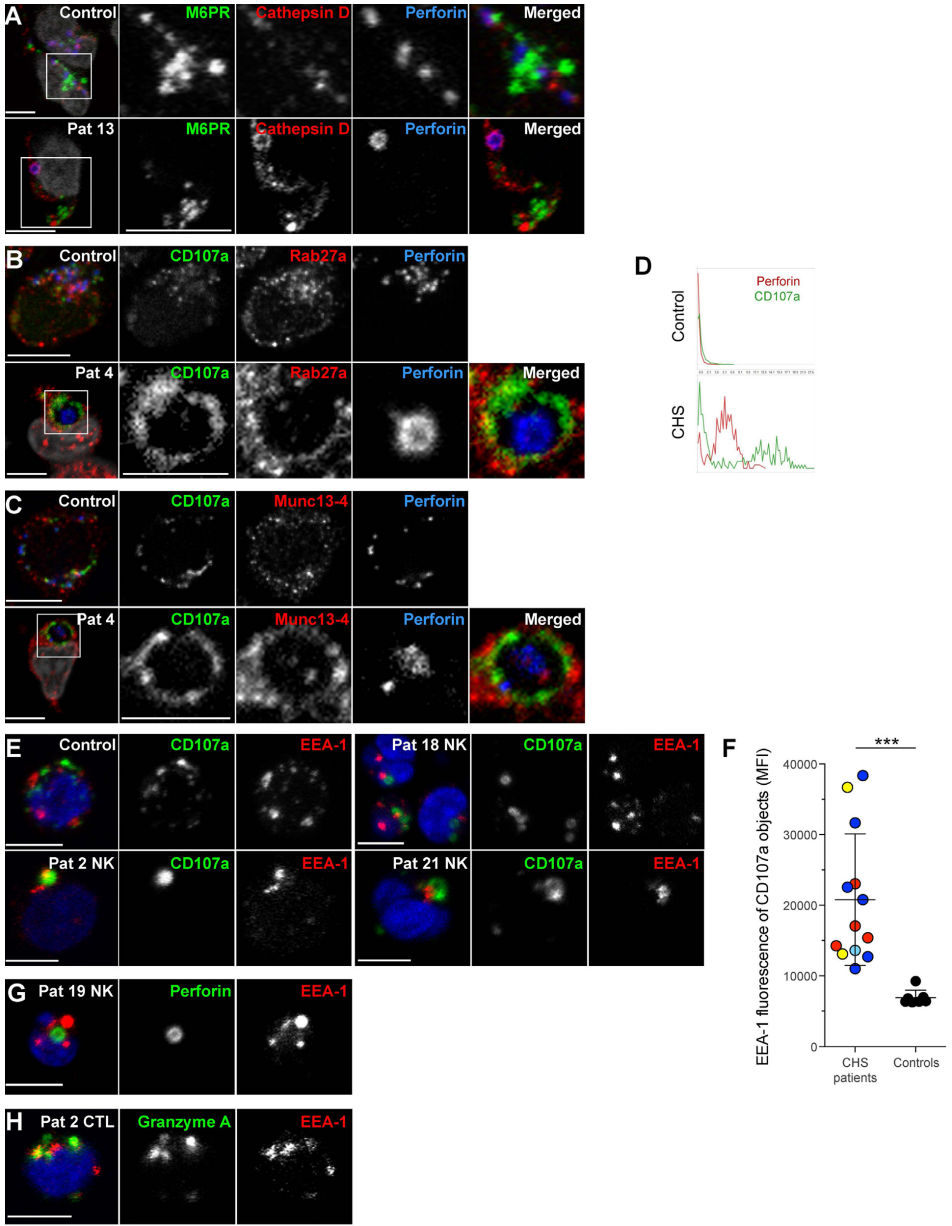
**Chediak–Higashi syndrome (CHS) NK cell granules are positive for Munc13-4, Rab27a, CD107a, cathepsin D, and EEA1, but not mannose-6-phosphate receptor**. Freshly isolated NK cells were labeled for perforin, DAPI and **(A)** M6PR and cathepsin D, **(B)** CD107a and Rab27a, or **(C)** CD107a and Munc13-4. **(D)** Traces show perforin and CD107a staining along a transect of a representative cytotoxic granule in a NK cell from (top panel) healthy control individual as well as (bottom panel) CHS patient, respectively. **(E)** Freshly isolated NK cells from a healthy control and three CHS patients with various *LYST* mutation positions were labeled for CD107a, EEA1, and DAPI, as indicated. **(F)** EEA1 median fluorescence intensity (MFI) within CD107a-positive objects was quantified in healthy control and CHS patient NK cells. On average 90 (range 8–187) CHS patient and 85 (range 42–135) control NK cells were quantified. **(G)** Freshly isolated NK cells from a CHS patient labeled for perforin, EEA1, and DAPI, as indicated. **(H)** Freshly isolated T cells from a CHS patient labeled for granzyme A, EEA1, and DAPI, as indicated. In micrographs, scale bars indicate 5 μm.

In order to further characterize the enlarged granules in CHS patient cells, freshly isolated NK cells were labeled for proteins to demarcate cytotoxic granules, late endosomes and early endosomes (Figure 6). Perforin and/or CD107a were used to label the giant lysosomal compartment. A hole was sometimes observed in the center of the perforin positive organelle in CHS NK cells, as previously noted in CTL (Figure 6D) (42, 43). The late endosomal/lysosomal enzyme cathepsin D localized to the lumen of the enlarged cytotoxic granules in CHS patient NK cells (Figure 6A) and has previously been observed to be processed and localized correctly in CHS cultured CTLs, as expected from the largely normal lysosomal function in most cell types of CHS patients (32, 43). However, NK cell cytotoxic granules from CHS patients were negative for mannose-6-phosphate receptor (M6PR, Figure 6A), which transfers some lysosomal enzymes, including granzymes and serglycin, from the trans-Golgi network to late endosomes (44, 45). M6PR partially colocalized with perforin granules in healthy freshly isolated NK cells. Notably, the cytotoxic granule exocytosis regulators Rab27a and Munc13-4 were found to decorate the cytotoxic granule limiting membrane in freshly isolated, unstimulated CHS NK cells (Figures 6B,C), contrasting healthy donor cells where Rab27a and Munc13-4 co-localization is specifically induced by signals from activating receptors (46, 47). Moreover, intriguingly, freshly isolated CHS NK cells and CD3^+^perforin^+^ cells showed EEA1 labeling of the enlarged lytic granules in all 13 patients examined (Figures 6E–H). Taken together, the present results extend earlier findings of mislocalized early and late endosomal markers on CHS lysosomes in cultured NK and T cells to freshly isolated cytotoxic lymphocytes.

## Discussion

Here, we studied a cohort of 21 CHS patients, evaluating cytotoxic T cell and NK cell lysosome morphology, exocytosis and clinical presentation while questioning whether any of these parameters correlated with each other or with specific *LYST* mutations. In addition, we studied vesicular markers with respect to cytotoxic granule identity, revealing novel aberrances in protein localization and sorting.

A key clinical challenge with CHS is to predict the likelihood of developing an accelerated phase of disease culminating in HLH. In our cohort, six adults with some degree of oculocutaneous albinism and neurological sequelae but no HLH episodes have at least one LYST allele of >3,000 amino acids which includes the ARM/HEAT, ConA-like and PH-like domains. The relatively mild disease in these adult patients thus suggests some residual LYST function in immune cells. In the absence of an antibody to LYST protein, it is unclear whether truncating mutations throughout the *LYST* gene result in partially functional LYST protein fragments, or whether such mutant proteins are degraded. In our assays, these patients displayed equally disrupted cytotoxic granule morphology and exocytosis as compared to patients that suffered an accelerated phase. Moreover, we did not find any correlations between mutation position of truncation mutants and cytotoxic granule morphology or lysosomal exocytosis. Notably, two early-onset HLH patients carried mutations toward the C-terminus of *LYST*, one in homozygous form (patient 14) and another in a compound heterozygous form (a point mutation and one truncation in the BEACH domain, patient 21). Our cohort also included two siblings discordant for presentation of HLH. Thus, patients with identical mutations may present with highly divergent clinical pictures (26), and the location of mutations in *LYST* cannot reliably predict development of HLH.

Jessen and colleagues previously described a stronger impairment of antigen-specific T cell exocytosis in *souris* relative to *beige* mice, which have an exon 27 splice site versus an exon 54, C-terminal single amino acid deletion in *Lyst*, respectively (20). While NK cells displayed uniformly defective exocytosis following engagement of YAC-1 cells, the degree of impaired T cell exocytosis and cytotoxicity corresponded to development of HLH in *souris*, but not *beige* mice. Congruently, in a cohort of 12 CHS patients, Jessen and colleagues found that late or no onset of HLH in CHS patients correlated with less impaired cytotoxicity of day 7 phytohemagglutinin (PHA)/IL-2 blast T cells, whereas fresh NK cell exocytosis was abnormal in all patients and, therefore, not predictive (20). This could indicate a differential role of LYST in the two cytotoxic lymphocyte populations. We, therefore, carefully compared the morphology and function of NK cells and CD8^+^CD57^+^ T cells, corresponding to *bona fide* antigen-experienced, cytotoxic T cells, in our cohort of CHS patients in order to determine any morphological or functional correlations. The results revealed that lymphocyte counts in patients tended to be elevated. Cytotoxic granule protein contents were comparable or elevated, though intracellular CD107a levels were reduced as compared to healthy controls. Remarkably, exocytosis was equally impaired among CTL and NK cells, from both HLH and non-HLH CHS patients, irrespective of cells being unstimulated or stimulated for 48 h with IL-2. In our analyses, the patients with the highest levels of exocytosis and recovery of function with IL-2 stimulation were those that had developed HLH. Contrasting the human T cell data from Jessen et al. (20), examining bulk PHA/IL-2 activated CD8^+^ T cells, our degranulation assays of fresh or short-term IL-2 stimulated CTL cells thus did not predict the severity of clinical outcome from CTL exocytosis. It is possible that differences in the stimulation protocol and the experimental read-out can explain these discrepancies. Our assays were robust, and revealed a significant increase in exocytosis upon strong receptor or cytokine-mediated activation of both CTL and NK cells.

Of note, patient 7 had over 10 LU NK cell cytotoxicity, which corresponded to the highest NK cell and CD8^+^CD57^+^ T cell exocytosis among the CHS patients (and within the normal range for healthy pediatric patients), although her sister (patient 8) displayed lower NK cell and CTL exocytosis as well as NK cell-mediated cytotoxicity. Patient 7 was sampled during an HLH flare, which can result in transiently elevated lymphocyte activity in FHL patients due to hypercytokinemia (48). Unfortunately, this patient succumbed to an accelerated phase of HLH at the age of 3 years. This observation serves as an additional note of caution with respect to interpreting increased cytotoxic lymphocyte exocytosis as a possible predictor of milder CHS.

The process of cytotoxic granule exocytosis requires formation of an IS, instigating DAG-gradient directed MTOC recruitment, trafficking of perforin-containing granules along microtubules to the MTOC, polarization of MTOC to the IS, granule migration along microtubules, and through an actin meshwork to finally fuse with the plasma membrane (49). CHS lymphocytes and other cell types contain normal numbers and lengths of assembled microtubules (50). Transport of CHS lysosomes along microtubules *in vitro* is possible, indicating correct localization of microtubule-interacting proteins; however, trafficking of such enlarged lysosomes in the cytoplasm may be spatially constrained (12, 35). Substantiating findings in CTL clones and IL-2 cultured NK cells (25, 42), we found that MTOC recruitment to the IS was impaired in freshly isolated CHS NK cells. Defective exocytosis, therefore, is apparently not solely due to enlarged granule size, as patients with *LYST* mutations that result in smaller granules did not demonstrate proportionately improved transport to the synapse or exocytosis. Moreover, we found that CHS patient CTL cytotoxic granules were on average less than half the diameter of NK cell granules, yet the exocytosis defect was equally severe in both cell types. These findings may suggest differences in regulation of lysosomal fusion and fission in NK cells versus CTL. Notably, melanosome secretion is also defective in CHS patients, a process that is not a polarized secretion event and requires granule movement only away from the MTOC.

Recently, defective granule exocytosis by CHS cytotoxic lymphocytes has been attributed to disrupted vesicular identity (25, 42). Unlike healthy NK cell cytotoxic granules, enlarged CHS granules did not contain M6PR. M6PR localized to secretory lysosomes in CD8^+^ CTL clones during early stages of culture, while granule biogenesis is underway, but not after 7 days of culture (32). In CHS B cell lines, M6PR is mis-sorted to enlarged lysosomes rather than the trans-Golgi network and late endosomes (13). Our data indicate that circulating CHS NK cells contain mature granules that no longer require delivery of nascent protein from the Golgi complex. Cytotoxic granule exocytosis is mediated by machinery including Munc13-4 and Rab27a, proteins recruited to cytotoxic granules only upon activation signals in both CTL and NK cells (46, 47). Substantiating findings in CTL clones and IL-2 cultured NK cells (25, 42), we found that enlarged CHS granules co-localized with Munc13-4 and Rab27a in freshly isolated NK cells. It is not clear if mistargeting of these facilitators of granule exocytosis can result in defective exocytosis. Overexpression of Munc13-4, Rab27a, and Slp3 could rescue the secretion defect in CHS CTL (42). Intriguingly, freshly isolated CHS NK cells and CD3^+^ cells showed that EEA1, an early endosome marker that regulates vesicle trafficking and fusion (51), overlapped with the enlarged cytotoxic granules in all 13 patients examined. Our observations contrast those of Sepulveda and colleagues (42), who did not find EEA1 (early endosome marker) colocalization with cytotoxic granules in CHS CTL clones, though Rab7 (late endosome marker) and Rab11 (recycling endosome marker) both mislocalized to granules. This discrepancy may reflect a difference between unstimulated lymphocytes freshly isolated from blood and CTL clones cultured in cytokines over several weeks. In non-immune cell lines, siRNA to *LYST* induces enlarged lysosomes but did not affect the size of early endosomes or result in localization of early endosome proteins to enlarged lysosomes (52). The presence of LYST during organelle biogenesis prior to the siRNA treatment may preserve early endosome morphology, or there may be a cell type-specific defect in immune cells or cells with specialized secretory lysosomes.

Both an excess of fusion and absence of fission have been hypothesized as the cause of the enlarged CHS lysosomes. Our data indicate that not only is there an excess of fusion between compartments that would normally fuse in the course of lysosome formation (53), but mistargeted fusion of early endosomes with these later compartments occurs, and this is not corrected by fission of membranes containing identifier of early endosomes. Interestingly, EEA1 did not coat the entire cytotoxic granule, but was confined to one portion of the granule, indicating a partial loss of organelle identity, possibly as proteins cluster but fail to fission off. As CHS NK cells had on average one enlarged cytotoxic granule relative to three enlarged cytotoxic granules in CTL, there may be differences in the regulation of cytotoxic granule biogenesis and fusion in different cytotoxic lymphocyte subsets. The molecular basis for such differences warrants further investigations.

In conclusion, we detail cytotoxic granule morphology and vesicular identity in cytotoxic lymphocyte subsets freshly isolated from peripheral blood. Our analyses uncover several aberrances with respect to CHS cytotoxic granule identity, as well as intriguing morphological differences between CTL and NK cells. Moreover, our results demonstrate similar impairments in CHS CTL and NK cell exocytosis, despite a more severe defect in granule morphology in NK cells. In terms of predicting susceptibility to developing HLH, it thus appears that differences between CTL and NK cell exocytosis may not be predictive. It is, however, possible that differences in exocytosis by activated cytotoxic lymphocyte may provide some guidance (26). Moreover, less severe missense mutations generally also appear associated with a lower susceptibility to develop HLH.

## Ethics Statement

This study was approved by The Regional Ethics Review Board in Stockholm (approval number 2006/228-31/3, 2006/229-31/3, 2006/230-31/3, 2008/1689-32). Peripheral blood from patients with suspected CHS was obtained with their/their parents’ written informed consent in accordance with the Declaration of Helsinki.

## Author Contributions

SC performed functional assays and wrote the manuscript; SMW performed microscopy and wrote the manuscript; BT performed genetic analyses; HA, WA-H, SA, FB, UC, ZK, KL, TP, HT, AU, and WI provided samples, clinical data, and cared for the patients; J-IH, MN, H-GL, MM, SE, and KK provided supervision and critically reviewed the manuscript; YB designed the study, interpreted results, and wrote the manuscript. All authors approve the final manuscript version.

## Conflict of Interest Statement

The authors declare that the research was conducted in the absence of any commercial or financial relationships that could be construed as a potential conflict of interest.
